# The association of sarcopenia and frailty in diabetes‐related foot disease: A 3‐year prospective evaluation

**DOI:** 10.1002/jfa2.70038

**Published:** 2025-03-22

**Authors:** Kay Yee Hon, Madeleine Bain, Suzanne Edwards, Guilherme Pena, Neil McMillan, Robert Fitridge

**Affiliations:** ^1^ Discipline of Surgical Specialties Adelaide Medical School The University of Adelaide Adelaide South Australia Australia; ^2^ Department of Vascular and Endovascular Surgery Royal Adelaide Hospital Adelaide South Australia Australia; ^3^ Basil Hetzel Institute for Translational Health Research The Queen Elizabeth Hospital Adelaide South Australia Australia; ^4^ School of Public Health Faculty of Health and Medical Sciences The University of Adelaide Adelaide South Australia Australia

**Keywords:** diabetes‐related foot disease, frailty, handgrip strength, sarcopenia, wound healing

## Abstract

**Aim:**

To prospectively evaluate the association of various markers of sarcopenia and frailty with clinical outcomes in diabetes‐related foot disease (DRFD), namely wound healing, amputation‐free survival, and death over 3 years.

**Methods:**

This was an observational study of patients with DRFD at a quaternary multidisciplinary diabetic foot service. Initial assessment includes classification of DRFDs using WIfI classification, assessment of frailty using the FRAIL scale, and measurement of handgrip strength (HGS) using a dynamometer. Muscle mass was ascertained by measuring the psoas muscle area at the level of L3 vertebrae on computed tomography. Patients were followed up for 3 years and primary outcomes were wound healing, amputation‐free survival, and death.

**Results:**

One hundred patients with a median age of 71 were included in the analysis. The majority of the patients were male (75%). Forty‐seven percent of patients were considered as frail, with 37 patients recorded to have low HGS. Patients with high HGS had significantly higher odds of wound healing by 3.83 times when compared to those with low HGS (odds ratio = 3.83. 95% CI 1.35–10.92). Patients with low psoas muscle index (PMI) and low HGS were observed to have a higher risk of death based on the following hazard ratios: HGS (high vs. low), HR = 0.46, 95% CI: 0.22–0.997; PMI (low vs. high), HR = 2.15, 95% CI: 1.17–3.96.

**Conclusion:**

There was a significant prevalence of frailty and reduced HGS among our patients with DRFD. Low HGS was associated with poor wound healing and increased mortality in patients with DRFD. Additionally, low muscle mass was associated with increased mortality in this population. This research highlights the need for more precise tests and future studies of the links between sarcopenia, frailty, and outcomes in DRFD.

**Abbreviations**
HGS: Handgrip strength
LLA: Lower limb amputation
PMI: Psoas muscle index
TPA: Total psoas muscle area

## 1 INTRODUCTION

The global point prevalence of diabetes‐related foot disease (DRFD) among people with diabetes has been reported to be 6.3% based on a comprehensive analysis of literature spanning the last 3 decades [1]. It is a highly comorbid complication of diabetes mellitus (DM) and is known to contribute to serious adverse health outcomes such as prolonged hospitalization, loss of functional independence, lower limb amputation (LLA), and death [2, 3]. In a recent 14‐year prospective review of patients with known DM, diabetes‐related foot ulcers are associated with more than seven times the increased risk of mortality when compared to those without ulcers, with a 5‐year and 10‐year mortality of 22% and 71%, respectively [4]. In Australia, the incidence of LLA due to DRFD is estimated to be between 5.2 and 7.2 per 1000 person‐years with diabetes) [5].

Given the serious implications of DRFD to mortality and morbidity of patients living with diabetes, the identification of factors associated with occurrence and healing of DRFD is needed to guide clinicians in providing adequate care to patients. There is a widespread interest in the role of sarcopenia and frailty in the aging population with DM, given that these elements are intrinsically related and have been proven to contribute to poor mortality and morbidity independently [6, 7, 8, 9, 10].

The relationship between sarcopenia, frailty, and diabetes is complicated as they often coexist and are related to other factors (especially aging), and evidence has shown that there are varying common and distinct mechanisms that contribute to the pathophysiology of these conditions. Sarcopenia is even regarded as ‘the intermediate step between diabetes and frailty’ [9, 10].

Frailty is a complex, dynamic, and multidimensional clinical entity that is the consequence of declined physiological reserve in multiple systems that increase patient’s vulnerability to stressors [11, 12, 13]. Frailty becomes more common with age, and our understanding of the underlying pathophysiology is incompletely understood and evolving. There are multiple postulated mechanisms, including chronic inflammation, mitochondrial dysfunction, cellular senescence, and hormonal changes that can contribute to the development of frailty [14, 15]. Frailty and DM are believed to share a common pathogenetic basis as risk factors associated with DM such as sedentary lifestyle and poor nutrition are linked with the development of frailty as well [8]. The coexistence of frailty and DM is common and is associated with increased severity of ischemia and tissue loss at the presentation of DRFD and a higher risk of mortality [7].

The European Working Group on Sarcopenia in Older People has defined sarcopenia as a clinical syndrome characterized by a ‘progressive and generalized loss of skeletal muscle mass and strength’ that contributes to the risk of adverse health outcomes [16]. Given that muscle mass is the largest storage of protein in the human body as well as its role as an insulin‐sensitive organ system, it plays a crucial role in protein metabolism, with direct effects on homeostasis and healing. For a diagnosis of sarcopenia to be made, patients must have objectively assessed low muscle mass and either poor muscle strength or performance. Sarcopenia is a predictor postoperative mortality and morbidity as well as development of cancer; however, there is limited studies into the effect of sarcopenia and frailty in DRFD [15, 17, 18, 19, 20].

Given a preponderance of related evidence of the role of sarcopenia and frailty in a variety of health factors, but limited evidence directly linking them to DRFD‐related wound healing and morbidity, our study aimed to assess the incidence of frailty and sarcopenia in patients with active DRFD and the influence of these factors on amputation‐free survival, complete ulcer healing, and death.

## 2 METHODS

### 2.1 Study design

This was a prospective observational study, with participant recruitment between February 2017 and December 2018 in three major South Australian tertiary hospitals. Patients with a confirmed diagnosis of DM and active foot ulceration who were either admitted to the Royal Adelaide Hospital or attended multidisciplinary foot clinic at Royal Adelaide Hospital, Lyell McEwin Hospital, or The Queen Elizabeth Hospital in Adelaide during this time were screened for suitability and recruited. Informed written consent was obtained from all participants and the study received ethics approval from the local institutional review board [Central Adelaide Local Health Network (CALHN) Ref: Q20161003, HREC Ref: HREC/16/TQEH/219].

At the time of recruitment, baseline demographic and clinical data were obtained. Specific baseline wound and limb status was assessed and categorized using the WIfI classification system [21, 22]. This risk stratification tool incorporates the status of the wound, degree of ischemia measured with toe pressures, and the presence of foot infection. Perfusion assessment was performed by a trained Doppler technician using manual photoplethysmography to measure toe pressure (mmHg).

Participants were followed up for up to 36 months where the outcomes of interest were time to healing of the index wound, wound healing status of index wound at 36 months, amputation‐free survival (defined as patient alive without the amputation of the index leg at the transtibial level or above), and death. Clinical and radiological markers of sarcopenia and frailty were evaluated for their impact on each of the four outcomes.

Two surrogate markers of sarcopenia were included in the study, namely muscle mass, assessed by both total psoas area (TPA) and psoas muscle index (PMI) and muscle strength, assessed using handgrip strength (HGS). Axial CT images obtained at the most caudal aspect of third lumbar vertebrae (L3) were identified and utilized for the measurement of TPA using the OSIRIX imaging software (Bernex, Switzerland). The area of psoas muscles was measured bilaterally using the measurement tool within the software. Mean TPA was obtained from the measurements performed by three observers, and interobserver reliability was then assessed using a Bland–Altman plot. PMI was then calculated by dividing the TPA with the square of the patient’s height in meters (TPA/height [2]). For our study, low muscle mass was identified by sex‐specific lowest quartile (Q1) of the PMI and TPA [19].

Handgrip strength was assessed using an isometric dynamometer (TTM Advanced Hand Dynamometer, Tokyo, Japan) following a standardized procedure. The mean of three measurements taken from the participants’ dominant hand was calculated and stratified by gender based on the EWGSOP2 recommended cutoff for low grip strength of <16 kg for female and <27 kg for male [16].

Frailty was screened using a validated questionnaire‐based tool to assess the five components of frailty: fatigue, resistance, multi‐morbidity, unintended weight loss, and poor ambulation [23]. Patients were asked to answer the following questions ‘yes’ or ‘no’ for each of the five components:

**Table:** 

Fatigue	Do you feel tired most/all of the time?
Resistance	Do you have difficulty walking up one flight of stairs without assistance?
Illness	Do you have more than 5 illnesses?
Weight loss	Have you lost more than 5% of your weight in the past year unintentionally?
Ambulatory	Do you have difficulty walking 1 km without assistance?

Each ‘yes’ was scored 1, with a score of ≥3 indicative of frailty.

### 2.2 Statistical methods

Fine and Gray competing risk models were used to investigate the association between various variables and time to wound healing and binary logistic regression for amputation‐free survival and wound healing status. Cox proportional models were used for evaluating time to death against each predictor. Each model was completed unadjusted. Various confounders that have been used in the previous study were then included in adjusted models, including WIfI clinical stages, age, body mass index (BMI) (<25 vs.>25), eGFR (<30 vs.>30 mL/min), HbA1C, previous amputation, and history of cardiovascular diseases (ischemic heart disease, heart failure, cerobrovascular disease, and peripheral arterial disease) [24]. A *p*‐value <0.05 was considered significant. Unadjusted ordinal logistic regression models were performed to investigate the association between WIfI stages and grip strength, TPA, PMI, sarcopenia, frailty, and cardiovascular disease. A multivariable ordinal logistic regression model was also performed for WiFI stages and the binary predictors namely frailty, sarcopenia, and cardiovascular system (CVS) disease. Kaplan–Meier curves were produced for each predictor and primary outcome. The statistical software used was SAS OnDemand for Academics (SAS Institute Inc. 2021) and Stata Statistical Software: Release 15.1 (College Station, TX: StataCorp LP).

## 3 RESULTS

### 3.1 Participants characteristics

A total of 184 patients were recruited; however, only 100 patients had adequate information to ascertain sarcopenia and frailty (Table 1).

**TABLE 1:** Baseline characteristics of 100 patients included in analysis.

Variable	Patients (*n* = 100)
Male, *n* (%)	75 (75%)
Age, years, median (IQR)	71 (17.8)
Diabetes duration, years, median (IQR)	14 (10)
HbA1c, %, median (IQR)	7.8 (2.6)
Insulin‐dependent, *n* (%)	40 (40)
Chronic kidney disease (CKD) stage 4, (eGFR ≤ 30), *n* (%)	10 (10)
Minor foot amputation, *n* (%)	23 (23)
Major limb amputation, *n* (%)	5 (5)
WIfI clinical stages, *n* (%)	
1	21 (21)
2	17 (17)
3	28 (28)
4	34 (34)
Body mass index (BMI), *n* (%)	
Underweight (<18)	3 (3)
Normal (18–25)	26 (26)
Overweight (25.1–30)	32 (32)
Obese (30.1)	39 (39)
Low grip strength, *n* (%)	39 (39)
Clinical frailty, *n* (%)	47 (47)
Sarcopenia, *n* (%)	16 (16)
Psoas muscle area, cm^2^, median (IQR)	22 (10.8)
Psoas muscle index, median (IQR)	7.3 (3)

*Note*: Age, duration of diabetes and Hba1c are presented as median (interquartile range). All other data are presented as raw numbers and percentages.

Amongst the 100 patients, 47 patients fulfilled the FRAIL scale criteria for frailty, whereas 16 were identified as sarcopenic, exhibiting low muscle strength and reduced muscle mass (Table 2). The main statistically significant differences between frail and nonfrail patients were in age (frail patients being older (*p* = 0.003) and higher proportion of females in the frail group (*p* = 0.05). There was a significantly higher proportion of nonfrail patients that had stage 4 chronic kidney disease (CKD) compared to frail patients (*p* = 0.04).

**TABLE 2:** Characteristics of patients according to frailty status.

Variable	Frail	Not frail	*p* value
Female (%)	16 (16)	9 (9)	0.05
Male,n (%)	31 (31)	44 (44)	0.23
Age, years, median (IQR)	75 (12.5)	66 (18)	0.003
Diabetes duration, years, median (IQR)	14 (10)	15 (13.25)	0.34
HbA1c, %, median (IQR)	7 (2.25)	8 (2.6)	0.56
Chronic kidney disease (CKD) stage 4, (eGFR ≤ 30), *n* (%)	2	8	0.04
Body mass index (BMI), *n* (%)			
Underweight (<18)	2 (2)	1 (1)	0.49
Normal (18–25)	14 (14)	11 (11)	0.42
Overweight (25.1–30)	14 (14)	19 (19)	0.66
Obese (30.1)	17 (17)	22 (22)	0.59
Low grip strength, *n* (%)	28 (28)	11 (11)	0.62
Sarcopenia, *n* (%)	11 (11)	5 (5)	0.09

*Note*: Age, duration of diabetes and Hba1c are presented as median (interquartile range). All other data are presented as raw numbers and percentages.

In this study population, 16 patients were identified as meeting the clinical criteria for sarcopenia, characterized by diminished muscle mass and strength (Table 3). Sarcopenic patients had a significantly longer duration of diabetes (median of 21 vs. 13 years, *p* = 0.04). There was also significant differences across all BMI categories—with sarcopenic group demonstrating higher proportion of normal weight and underweight individuals and lower proportion of overweight and obese individuals.

**TABLE 3:** Characteristics of patients between sarcopenic and nonsarcopenic patients.

Variable	Sarcopenic	Not sarcopenic	*p* value
Female	5 (5)	20 (20)	0.53
Male	11 (11)	64 (64)	
Age, years, median (IQR)	72 (16)	71 (16)	0.76
Diabetes duration, years, median (IQR)	21 (19)	13 (10.5)	0.04
HbA1c, %, median (IQR)	7.1 (1.9)	8.0 (2.6)	0.77
Chronic kidney disease (CKD) stage 4, (eGFR ≤ 30), *n* (%)	3 (3)	7 (7)	0.43
Body mass index (BMI), *n* (%)			
Underweight (<18)	2 (2)	1 (1)	0.01
Normal (18–25)	8 (8)	17 (17)	0.01
Overweight (25.1–30)	3 (3)	30 (30)	0.01
Obese (30.1)	3 (3)	36 (36)	0.01

*Note*: Age, duration of diabetes and Hba1c are presented as median (interquartile range). All other data are presented as raw numbers and percentages.

At the 36‐month follow‐up assessment, 60 patients achieved complete wound healing, whereas 25 patients did not achieve wound closure, and 15 patients required further amputation. At the end of the follow‐up, 45 patients had died.

The psoas muscle area was assessed by three observers, and intraclass correlation coefficients were 0.982, 95% CI 0.88–0.99. We found a week correlation between handgrip strength and PMI with an *R* value of 0.253 (*p* = 0.01).

### 3.2 Wound healing status

Grip strength in the dominant hand, when analyzed with both continuous and binary data, was shown to have an association with wound healing status. For each 1 kg increase in grip strength, the odds of having the wound healed was 5.7% greater (OR = 1.057, 95% CI: 1.006–1.111). When evaluated as binary data, patients with high grip strength have higher odds of wound healing by 3.83 times when compared to those with low grip strength (OR = 3.83, 95% CI 1.35–10.92). There was no statistically significant association between wound healing status with frailty (*p* = 0.37), total psoas muscle area (*p* = 0.35), or PMI (*p* = 0.23).

### 3.3 Time to wound healing

**FIGURE 1:**
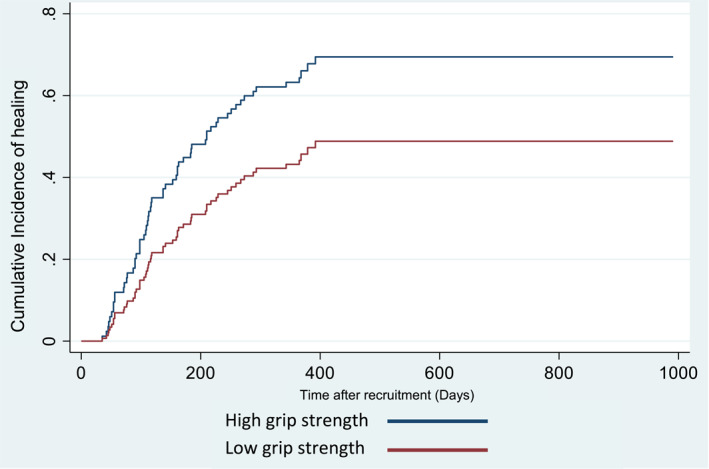
Cumulative incidence function (CIF) for time to wound healing by handgrip strength (low or high).

There was a statistically significant association between time to wound healing and grip strength in the dominant hand when adjusted for confounding factors (WIfI clinical stages, age, BMI, eGFR, HbA1c, and previous LLA). For a one unit (1 kg) increase in grip strength, the subdistribution hazard of wound healing was 4% greater (subdistribution hazard ratio = 1.04, 95% CI: 1.001–1.07) (Figure 1). There was no statistically significant association between time to wound healing with frailty, total psoas muscle area, or PMI.

### 3.4 Amputation‐free survival

There was no statistically significant association between amputation‐free survival and any of the predictors whether unadjusted or adjusted for confounding factors.

### 3.5 Death

Forty‐five deaths were recorded, with only 22 cases having documented cause of death. Cardiovascular diseases accounted for 6 deaths, 9 deaths were related to cancer diagnosis, 4 related to sepsis, and 4 deaths were accountable to respiratory illness. Amongst 45 deaths, 26 (57.8%) were frail.

**FIGURE 2:**
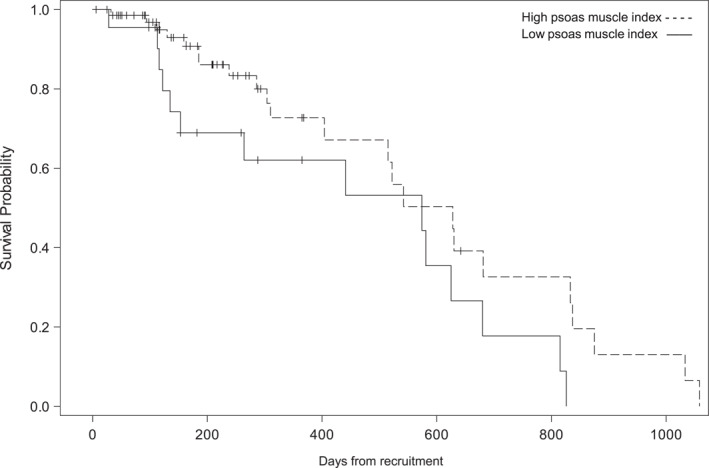
Kaplan–Meier survival curves showing the association between the psoas muscle index and time to death.

**FIGURE 3:**
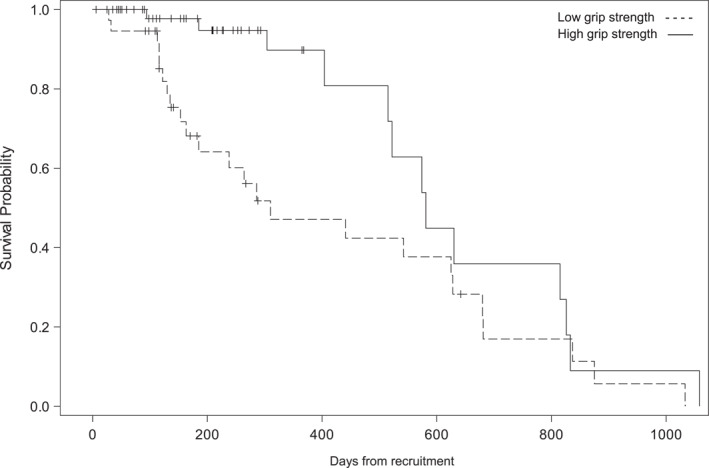
Kaplan–Meier survival curves showing the association between handgrip strength (HGS) and time to death.

A statistically significant association was found between time to death and PMI (unadjusted *p* = 0.0426). Patients with a low PMI had a hazard of dying 1.93 times greater than patients with a high PMI (HR = 1.93, 95% CI 1.02, 3.68) (Figure 2). In an adjusted model, there was no statistically significant association between time to death and PMI (adjusted *p* = 0.118). However, with degrees of freedom = 8 (8 covariates), there was likely an issue of sparse data (=45 deaths). There was also an association with time to death and grip strength when adjusted for confounding factors (adjusted *p* = 0.0490) (Figure 3). Patients with high grip strength had a hazard of death 54% less than patients with low grip strength (HR = 0.46, 95% CI: 0.22, 0.997).

### 3.6 WIfI stages

There were no statistically significant associations between WIfI stages with frailty, HGS, or psoas muscle mass (PMM). However, there was a statistically significant (*p* = 0.005) association between WIfI stages and CVS disease adjusting for frailty and sarcopenia (adjusted *p* value = 0.008). Those with a history of cardiovascular disease had odds of high WIfI scores when compared to those without the history of cardiovascular disease (Odds Ratio = 3.55, 95% CI: 1.39, 9.05) (Table 4).

**TABLE 4:** Ordinal logistic analysis of association between handgrip strength, total psoas muscle area, psoas muscle index, and frailty with WIfI clinical stages.

Predictor	Odds ratio (95% CI)	*p*‐value
Hand‐grip strength (in kg)	1.01 (0.98, 1.05)	0.4474
Hand‐grip strength (low vs. high)	0.85 (0.41, 1.73)	0.6488
Total psoas muscle area (per 1 cm^2^ increase)	0.98 (0.93, 1.03)	0.4332
Psoas muscle index (per 1 unit increase)	0.93 (0.78, 1.11)	0.4314
Total psoas muscle area (low vs. high)	1.33 (0.58, 3.02)	0.4976
Psoas muscle index (low vs. high))	0.92 (0.41, 2.05)	0.8341
Frailty (yes vs. no)	1.92 (0.94, 3.95)	0.0745
Sarcopenia (yes vs. no)	1.11 (0.43, 2.90)	0.8268
Cardiovascular disease (yes vs. no)	3.74 (1.48, 9.43)	0.0052

*Note*: Modeling the probability that the WIfI stage is high (clinical stage 3 or 4).

## 4 DISCUSSION

This prospective observational study provided insights into clinical outcomes for patients with DRFD over a 3‐year period in addition to evaluating the association of frailty and sarcopenia with DRFD. We found that patients with DRFD and higher HGS had four‐fold higher odds of achieving wound healing when compared to patients with lower HGS. Additionally, there was also a 4% increased chance of faster wound healing for patients with each 1 kg stronger handgrip. Our analysis also suggested that low muscle mass was associated with higher mortality in patients with DRFD.

The significantly higher age and proportion of female in the frail group align with established literature showing that frailty prevalence increases with age and studies indicating that women are more likely to be frail than men [25, 26]. The higher prevalence of stage 4 CKD in the nonfrail group was unexpected as it is contradicting the evidence that advanced CKD is associated with higher frailty risk and earlier age of onset of frailty compared to those with a normal renal function [27, 28]. However, given the small number of patients with stage 4 CKD in the study (*n* = 10), there is concern regarding the potential for effect size inflation and the extent to which this sample adequately represents the broader population of patients with stage 4 CKD and diabetes‐related foot ulcers.

There was an association of longer diabetes duration and lower BMI in sarcopenic patients in this study population. A meta‐analysis of 46 studies found that the duration of diabetes was a significant risk factor for sarcopenia in adult patients (OR 1.06), which supports our findings [29]. The underlying pathophysiology may be attributed to the effects of insulin resistance and oxidative stress which impairs muscle synthesis and promotes proteins degradation, thereby contributing to the development of sarcopenia. Underweight BMI has been explored as a risk factor for the development of sarcopenia (OR 2.25) which aligns with our findings (Curtis, 2023). Higher BMI was a protective factor for the development of sarcopenia. However, in obesity states, the risk of sarcopenia is inconsistent, with reported differences when assessing obesity using percentage body fat compared to BMI measurement [30, 31].

Our cohort had a survival rate of 55% after 3 years of follow‐up for patients with a diagnosis of DRFD. A systematic review of 27 observational studies reported a survival rate of 66.9% (95% CI 59.3%–75.5%) at 3 years [32]. Cardiovascular diseases were noted to account for half of the deaths reported in the review. A high mortality rate of 64% was also observed in a study of 91 patients with DRFD followed up for 11 years, with a mean time to death of 5 ± 3 years [33]. The consistently high mortality rate associated with DRFD across different studies further emphasizes the significant burden of this disease and the need to identify and address risk factors that contribute to the poor clinical outcomes.

Our results suggested a trend of higher mortality rates in patients with low PMI when compared to those with high PMI, although this association did not reach statistical significance in the multivariate analysis. A similar observation was noted in a study involving 97 patients where low PMI have a significantly higher mortality rate following AKA for critical limb ischemia, despite no differences in age or discharge status. Additionally, sarcopenia has also been found to be a prognostic factor for overall survival in patients with critical limb ischemia (HR, 3.22; 95% CI, 1.24–9.11; *p* = 0.02) [34]. Another cohort study of 167 patients in South Korea showed a 5‐year mortality rate of 52.7% with sarcopenic patients having a higher mortality rate (60.7%) compared to those without (36.4%). Kaplan–Meier analysis confirmed these differences were statistically significant. Additionally, age and cardiac diseases emerged as independent predictors of survival, suggesting that sarcopenia interacts with broader health factors. This highlighted the critical impact of sarcopenia on long‐term survival following diabetes‐related LLA [15].

Handgrip strength is the preferred muscle strength parameter for the diagnosis of sarcopenia in Australia and New Zealand [35]. By using a dynamometer, HGS directly measures the functional status of the upper limb muscles, but it also indirectly acts as a surrogate marker for many health‐related risk factors and outcomes. Grip strength has been found to be a strong predictor for endurance, cancer, cardiovascular risk, and mortality in various health conditions including but not limited to heart failure, cancer, and DM [36, 37, 38, 39, 40]. In our study, we found that high HGS was associated with higher odds of wound healing and also faster wound healing. The underlying mechanisms leading to low grip strength also likely contributes to poor wound healing, as low HGS itself is unlikely to cause poor wound healing. Further research is needed to validate these findings and how practitioners could incorporate HGS assessments into routine clinical practices in the care of patients with DRFD.

We did not find a significant association of TPA and PMI with wound healing status or amputation‐free survival. Additionally, our study also did not demonstrate an association of low muscle mass with the severity of DRFD using WIfI classification. This is in contrast with the findings from a cross‐sectional study by Cheng et al. (2017), which found that sarcopenia is independently associated with DRFD, where sarcopenic patients had more foot ulcers, higher Wagner grade, and greater percentage of amputation when compared to patients without sarcopenia [41]. This discordance could be due to the different methods used to assess low muscle mass and severity of DRFD. In the study by Cheng et al, the skeletal muscle index using dual‐energy X‐ray‐absorptiometry (Dual energy X‐ray absorptiometry) was used, compared to our use of CT‐based measurement of PMM in this study. This discrepancy indicates the need for standardized methods to assess sarcopenia, particularly muscle mass, in the study of DRFD to ensure consistent reporting effects of sarcopenia with outcomes of DRFD [41, 42].

The assessment of muscle quantity or mass is pivotal to the diagnosis of sarcopenia, and there are various noninvasive imaging techniques currently available to directly or indirectly measure skeletal muscle mass [43]. Dual energy X‐ray absorptiometry allows measurement of bone mineral density as well as lean body and fat mass. Magnetic resonance imaging and CT are also considered as gold standards. Both imaging techniques allow quantification of muscle mass and/or volume using cross‐sectional images. Magnetic resonance imaging has superior ability to further ascertain intramuscular fat content, whereas CT is more easily accessible. Cutoff points for low muscle mass are not yet well defined for these measurements given the difference in patient populations being studied (oncological patients, geriatric populations, and vascular surgery) [16, 18, 20, 42, 44, 45]. Additionally, studies that attempted to ascertain population‐based cutoffs for PMI or psoas muscle area have different age ranges compared to our study [46]. Poor agreement in the literature limited our ability to select precise cutoffs for our sarcopenia indices.

We chose PMI and mass due to the availability of CT imaging for this patient population, who often required the assessment of underlying vascular diseases for their foot wounds using CT Angiography. However, a small prospective cohort study found no correlation between low psoas muscle area determined on CT compared to low muscle mass determined on Dual‐energy X‐ray absorptiometry and total skeletal muscle area on CT; nonetheless, low PMI was able to predict 1‐year mortality in a cohort of patients undergoing vascular surgery [42]. This may explain the lack of correlation between PMI and PMM in wound healing status, time to wound healing, and amputation.

Frailty, a multidimensional syndrome, is characterized by reduced physiological reserve, rendering individuals more susceptible to stressors. This vulnerability leads to prolonged recovery times, or in some cases an inability to fully recover from illnesses. It has been consistently shown to be associated with poor clinical outcomes across all domains of physical and mental health including increased risk of falls, impaired mobility, prolonged hospitalization, diminished quality of life, depression, and cognitive decline [11, 47, 48, 49]. In a systematic review by Fernando et al., frailty is found to be associated with more advanced disease presentation (more severe ulceration, infection, or ischemia), delayed wound healing, and increased risk of prolonged hospitalization, hospital readmission, amputation, and mortality [7]. Unexpectedly, no association was found between frailty and clinical outcomes examined in this study and this is likely due to the methodological approach used to define frailty.

There are various methods to assess frailty in clinical and research settings, each with its own strengths and weaknesses. The frailty phenotype (Fried’s criteria) is one of the most widely used methods [47]. It uses five criteria: unintentional weight loss, weakness (low grip strength), exhaustion, slow walking speed, and low physical activity as markers for frailty. It is primarily based on objective assessments which may provide more reliable and standardized results across different settings. The main obstacle to using the frailty phenotype in this study was the challenge of assessing walking speed in our patient cohort. Many patients presented with foot deformities were wearing offloading devices, which could complicate the interpretation of walking speed measurements as it would be difficult to distinguish if the reduced walking speed is due to frailty or caused by the foot deformities or offloading footwear. The FRAIL scale used in this study is a simple and practical tool to ascertain deficits in health and functional ability. It lacks the detailed physical performance metrics of the frailty phenotype; however, its simplicity and effectiveness in quickly identifying frailty has made it a practical choice in screening for frailty in the outpatient setting. However, the questionnaire‐style approach is heavily dependent on self‐reported information which could be influenced by patient’s perception, mood, or cognitive state. Unfortunately, both Fried’s phenotype and FRAIL scale lack the ability to capture cognitive and psychosocial aspects of frailty, which are important and understudied domains of frailty. A frailty prevalence of 74% was previously reported in a small prospective cohort study of 76 hospitalized patients with diabetes‐related foot ulceration (DRFU) using the frailty index [50]. Only 47% of our studied patients fulfilled the criteria for frailty, which, while high overall, is still relatively lower than observed in other studies. This difference may be explained by the different assessment methods of frailty in addition to the different clinical settings where patients were recruited. Patients in our study also included those who have attended outpatient clinics and may have been less likely to be frail given their ability to travel to these appointments when compared to those who were admitted in the hospital. As a result, the prevalence of frailty may be underestimated or overestimated depending on the chosen tools and study cohorts.

## 5 STRENGTH AND LIMITATIONS

A core strength of our study was its prospective design and long follow‐up period, which allowed for collection of detailed baseline and follow‐up outcome data. This was reflected in gathering both grip strength and muscle mass instead of focusing on a single measure, and, likewise, in gathering multiple outcomes, which improved the value of the dataset for each patient whom we were able to include in analyses. This highlights the need for future studies in this area to gather robust data over long durations as both are key to understanding outcomes in this cohort.

The study was limited by a relatively small sample size for the complexity of analyses; while the sample size was reasonable for a longitudinal study, the comorbidity and complexity of the patients (and the limitations on how many had adequate imaging to be included) in this cohort will likely need larger, multicenter studies to address broader implications for the relationship between sarcopenia and DRFD. This is especially true given the paucity of reliable and widely accepted validated measures of sarcopenia and frailty, which limits the precision of prognostic risk assessment in this cohort. Specifically, the development or adoption of standardized measures of sarcopenia and frailty in DRFD patients should be considered to allow a consistent and reliable assessment that would be of direct relevance to clinic practice.

Our study population was predominantly male, which may limit the generalizability of the results to female patients. This is not a surprising finding, as it is known that the incidence of DRFU is higher in men than women with a diagnosis of DM [2, 51]. Mortality was also reported to be higher in men than women in a previous sensitivity analysis [32], but this was contradicted by a large observational study that found women have lower survival probability following admission for DRFD [51].

## 6 CONCLUSION

This prospective cohort study demonstrated the interplay between measures of sarcopenia and frailty with patients in DRFD. We found a high prevalence of frailty and low grip strength in patients with DRFD and that low grip strength was associated with poor wound healing and mortality. Despite not finding an association between PMM with wound healing, we found a higher mortality rate in patients with low PMI.

However, there is a need for a consensus within the clinical and research communities on the most appropriate tools to assess sarcopenia and frailty in patients with DRFD. Uncertainties and inconsistencies remain in the literature about the association of sarcopenia and DRFD, which may be due to the lack of sensitive assessment tools to allow a reliable comparison between studies. To address this, considerations should be given for multicenter, national, or international studies with interdisciplinary collaborations to form a large dataset which may be able to provide more definitive insights into the association of sarcopenia and frailty in DRFD.

Additionally, there is also a need to further evaluate how clinicians and research can best translate these research findings into clinical practice to allow early identification and management of patients with low grip strength and/or low PMI which has been demonstrated to contribute to poor clinical outcomes. Incorporating grip strength assessment into screening of patients with DRFD could help identify patients at risk of poor wound healing and mortality. Nutritional support and exercise programs should also be considered to improve muscle mass and strength that could potentially enhance clinical outcomes for patients with diabetes and foot wounds.

## AUTHOR CONTRIBUTIONS

**Kay Yee Hon:** Data Curation; investigation, visualization, writing—original draft, writing—review and editing. **Madeleine Bain:** Investigation, writing—original draft, writing—review and edition. **Suzanne Edwards:** Formal analysis, visualization. **Guilherme Pena:** Conceptualization, data Curation, investigation, methodology, writing—review and editing. **Neil McMillan:** Project Administration, visualization, writing—review and editing. **Robert Fitridge:** Conceptualization, methodology, supervision, writing—review and editing.

## CONFLICT OF INTEREST STATEMENT

The authors declare no conflicts of interest.

## ETHICS STATEMENT

The study was approved by CALHN Human Research and Ethics Committee [CALHN Ref: Q20161003, HREC Ref: HREC/16/TQEH/219].

## Data Availability

The data that support the findings of this study are available from the corresponding author upon reasonable request.
